# Highly sensitive amplicon-based transcript quantification by semiconductor sequencing

**DOI:** 10.1186/1471-2164-15-565

**Published:** 2014-07-05

**Authors:** Jitao David Zhang, Tobias Schindler, Erich Küng, Martin Ebeling, Ulrich Certa

**Affiliations:** Roche Pharmaceutical Research and Early Development, Department of Pharmaceutical Sciences/ Translational Technologies and Bioinformatics, Roche Innovation Center Basel, Grenzacherstrasse 124, 4070 Basel, Switzerland

**Keywords:** Semiconductor RNA sequencing, Digital transcript imaging, Multiplex analysis

## Abstract

**Background:**

In clinical and basic research custom panels for transcript profiling are gaining importance because only project specific informative genes are interrogated. This approach reduces costs and complexity of data analysis and allows multiplexing of samples. Polymerase-chain-reaction (PCR) based TaqMan assays have high sensitivity but suffer from a limited dynamic range and sample throughput. Hence, there is a gap for a technology able to measure expression of large gene sets in multiple samples.

**Results:**

We have adapted a commercially available mRNA quantification assay (AmpliSeq-RNA) that measures mRNA abundance based on the frequency of PCR amplicons determined by high-throughput semiconductor sequencing. This approach allows for parallel, accurate quantification of about 1000 transcripts in multiple samples covering a dynamic range of five orders of magnitude. Using samples derived from a well-characterized stem cell differentiation model, we obtained a good correlation (r = 0.78) of transcript levels measured by AmpliSeq-RNA and DNA-microarrays. A significant portion of low abundant transcripts escapes detection by microarrays due to limited sensitivity. Standard quantitative RNA sequencing of the same samples confirms expression of low abundant genes with an overall correlation coefficient of r = 0.87. Based on digital AmpliSeq-RNA imaging we show switches of signaling cascades at four time points during differentiation of stem cells into cardiomyocytes.

**Conclusions:**

The AmpliSeq-RNA technology adapted to high-throughput semiconductor sequencing allows robust transcript quantification based on amplicon frequency. Multiplexing of at least 900 parallel PCR reactions is feasible because sequencing-based quantification eliminates artefacts coming from off-target amplification. Using this approach, RNA quantification and detection of genetic variations can be performed in the same experiment.

## Background

Personal healthcare has the goal to deliver specific targeted therapeutics to patient groups stratified based on predictive genetic or transcriptional markers. The availability of several hundred complete human genomes in the public domain has added a high degree of confidence to genetic markers predicting susceptibility to certain disorders. Robust and validated PCR-based assays interrogating disease specific single-nucleotide-polymorphisms (SNP) panels are routinely used in exploratory and clinical research using formalin fixed-paraffin embedded (FFPE) or biopsy derived DNA samples. The *de novo* discovery of dynamic mRNA biomarkers that inform about drug response or specific gene expression patterns is regularly done by genome-wide transcript profiling using DNA microarrays or quantitative RNA sequencing. However, both technologies require relatively high amounts of high quality RNA complicating genome-wide transcript profiling of clinical samples.

For translational research novel primary cell based tissue models are emerging. Induced pluripotent stem cell (iPSC) technology allows *in vitro* generation of tissue specific human cells such as cardiomyocytes. In addition, spherocyte based three-dimensional culture systems allow small-scale engineering of human tissues containing cell types present in the native tissue. In contrast to patient derived tissues these *in vitro* models can be exposed to drugs at various dose-levels and multiple time-points in a well-controlled laboratory environment. The main purpose of such primary cell based *in vitro* systems is analysis of drug-responses using a variety of dynamic parameters such as transcript levels. Differentially expressed transcripts found in 3D-cultures, human primary cells or clinical samples are useful resources for generation of customized assays interrogating expression of disease relevant genes only. The microfluidics based quantitative PCR (qPCR) platform (“Fluidigm”) allows multiparallel expression analysis of 96 custom transcripts in 96 samples at high sensitivity with low RNA input [1]. The Luminex system is a multiplexed color-coded microsphere-based suspension platform for digital quantification of up to 100 custom transcripts in a single tube [2]. The Nanostring technology uses complementary probes coupled with large color-coded DNA molecules to label transcripts followed by confocal microscope-based digital quantification at single molecule sensitivity [3]. Alternatively, oligonucleotide probe coated cantilever arrays have been used to quantify transcripts in cell lysates based on nano-mechanical bending [4]. The production of custom microarrays was discontinued by most suppliers due to high costs and declining market size. For routine applications, qPCR profiling (Taqman assay) is the method of choice because it is performed in conventional 96-well plates using standard thermocyclers [5]. In addition, custom gene qPCR panels covering various major biological processes such as apoptosis, cell cycle control or immune stimulation are commercially available from a number of vendors. All technologies briefly outlined above suffer either from low sample throughput or else from a rather limited number of transcripts for multiparallel analysis.

Recently a combination of PCR amplification and semiconductor sequencing termed AmpliSeq became commercially available for customized detection of sequence based single-nucleotide polymorphisms (SNPs) or point mutations [6]. Compared to standard PCR assays this technology provides the sequence flanking the mutation of interest at high coverage permitting quantitative determination of allele frequencies. For cancer research for instance, a commercial panel of 190 primer pairs covering hot-spot mutations in 46 cancer-related genes is available for low-throughput semiconductor sequencers [7, 8]. This gene panel was used in a pilot study for AmpliSeq validation using clinical (FFPE) lung cancer samples [9–11]. The amplicon size of 80 to 100 base pairs is compatible with partially degraded material which is very challenging to sequence by classical DNA sequencing methods such as Sanger-sequencing. All clinical FFPE samples were successfully amplified and sequenced with 10ng DNA input. The allele frequencies determined by Sanger-sequencing were confirmed by AmpliSeq technology [12]. Therefore it is likely that focused parallel sequencing will gain attention in clinical studies and for patient stratification.

Here we present the first AmpliSeq-based RNA quantification (AmpliSeq-RNA) in multiple samples using a custom panel of 917 individual amplicons quantified by high-throughput Ion-Torrent-Proton semiconductor sequencing [13]. For validation, we have chosen RNA samples derived from four time points of a published cardiomyocyte differentiation experiment as a well-characterized biological system [14]. Expression values measured by AmpliSeq-RNA data correlate well with equivalent microarray data or conventional quantitative RNA sequencing. Adaptation of AmpliSeq-RNA technology to high-performance semiconductor technology closes an important gap in custom RNA analysis because it allows for the first time multi-parallel expression analysis of hundreds of genes in up to one hundred samples covering a dynamic range of five orders of magnitude.

## Results

### Workflow of AmpliSeq-based digital transcript imaging (AmpliSeq-RNA)

**Figure 1:**
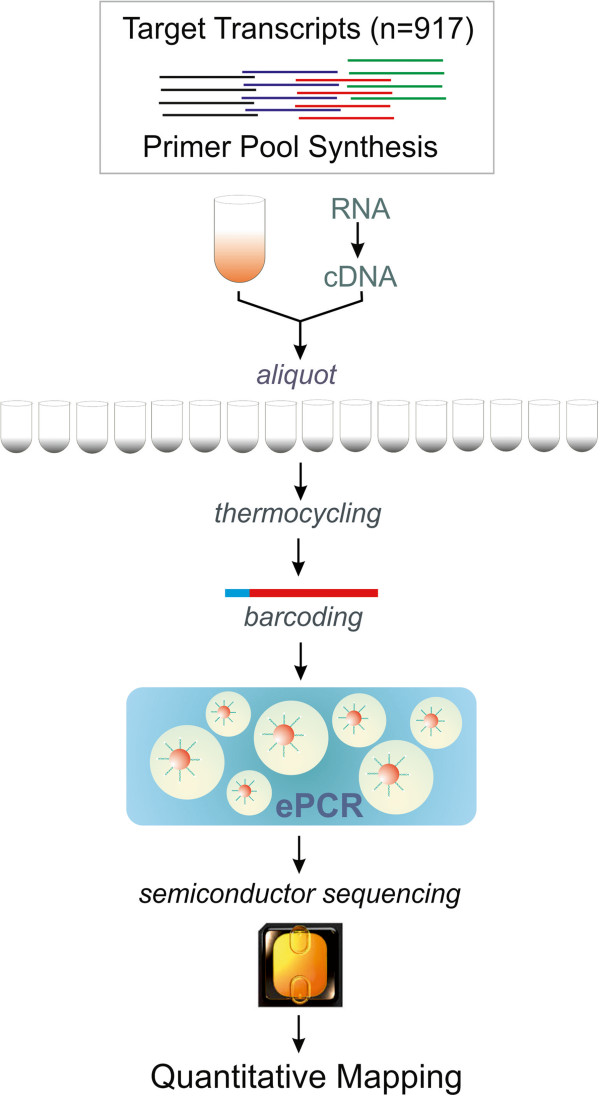
**Experimental workflow for AmpliSeq-RNA based transcript quantification.** An aliquot of the custom primer pool, the cDNA sample and a PCR master-cocktail are combined in individual tubes or multititer plate wells. Following pre-amplification the amplicons are tagged with a sample specific molecular barcode followed by pooling and emulsion PCR (ePCR) amplification on nanosphere-beads. After semiconductor sequencing (Ion-Torrent-Proton) reads are mapped to the target sequence library. Read frequencies proportional to transcript abundance are provided in a standard spread-sheet for further analysis (for experimental details see Methods Section).

Compared with DNA genotyping, the AmpliSeq-RNA quantification workflow (Figure 1) requires polyA-primed conversion of mRNA to cDNA followed by target specific PCR amplification using a multiplex primer pool. Each primer pair amplifies a unique 80-100 base pair cDNA fragment using a limited number of PCR cycles important for maintenance of transcript stoichiometry. After amplification a DNA-barcode is attached to each sample followed by pooling and emulsion-PCR of all samples. Following bead library construction the amplicon library is sequenced on an Ion-Torrent Proton instrument. Decoding of samples and quantitative mapping to the input amplicon sequences concludes the experimental workflow. For experimental validation of AmpliSeq-RNA we have compiled a custom panel of 917 transcripts representing about 150 biological signaling cascades. This gene set was collected from commercial pathway panels, public domain resources such as Pathway Commons (http://www.pathwaycommons.org) or relevant literature [Zhang, J.D., manuscript in preparation]. We anticipated that this pathway-based transcript panel would report major variances in gene expression programs across very diverse cell-types. Therefore we considered the differentiation of inducible pluri-potent stem cells (iPSC) into cardiomyocytes as an appropriate test case [14].

In addition, we adapted the AmpliSeq-RNA technology to the high-throughput Ion-Torrent Proton semiconductor sequencer which generates about 100-times more reads than the Personal Genome Sequencer (PGM) originally used. This expansion of capacity allows adaptation to the experimental needs by either adjusting the dynamic range or the number of samples. In the current study, the pooled samples coming from biological triplicates of four time-points (day 0, day, 10, day 20, day 60) were sequenced in parallel on a single chip. We obtained in average 16’362’403, 11’145’049, 15’951’406 and 12’467’509 mapped reads for day 0, day 10, day 20 and day 60 respectively. The most abundant amplicons of our panel are represented by up to 800’000 reads exceeding the needs of most profiling experiments. We have set the lower detection limit to an average of five reads per amplicon in order to reduce artefacts known as the “Monte Carlo effect” caused by stochastic single molecule amplification at low input RNA levels [15].

### Technical and biological validation of AmpliSeq-RNA

To keep this technology-focused study in a manageable scope we selected triplicate RNA samples from day 0, 10, 20 and 60 of our published iPSC differentiation experiment for AmpliSeq-RNA validation. To confirm integrity of the reference samples we have repeated the published microarray profiling analysis [14]. Prior to the biological evaluation we assessed technical variation of the AmpliSeq-RNA technology adapted to Proton-sequencing. We selected a single RNA sample (day 60) and prepared four independent sequencing libraries using 25 nanogram input RNA per technical replicate. Following sequencing and transcript quantification we calculated a common coefficient of variation of 0.16 (defined as sigma/mean) for AmpliSeq-RNA. The observed inter-sample variation is in the range of established RNA profiling technologies such as qPCR (1% ~ 15%; [16, 17]) microarray (5% ~ 15%; [18]) or quantitative RNA sequencing (10% ~ 15%; [19]).

### Comparison of AmpliSeq-RNA gene expression with microarrays and quantitative RNA-sequencing

**Figure 2:**
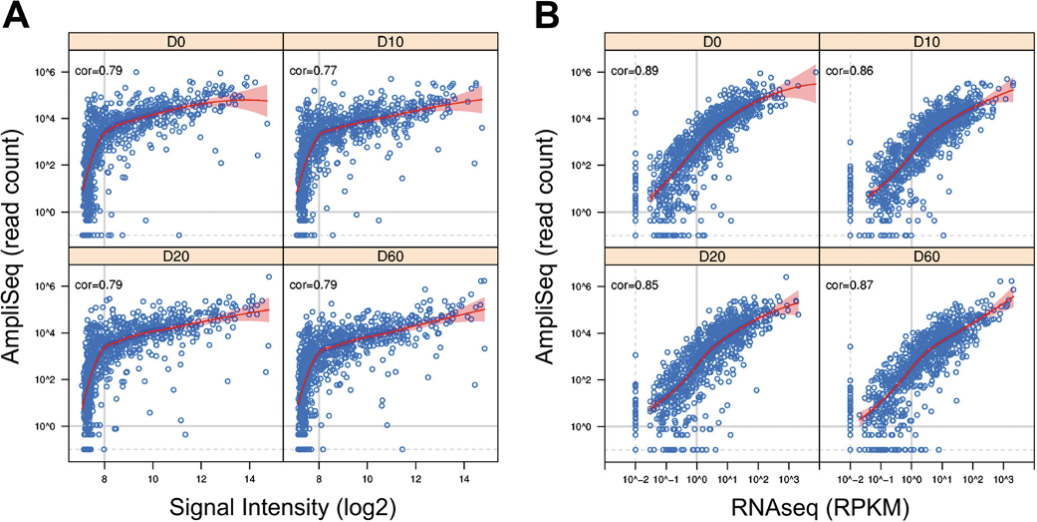
**Comparison of RNA- profiling to microarrays and quantitative RNA sequencing. (A)** Correlation of gene expression profiles generated by AmpliSeq-RNA (y-axis) and DNA microarrays (x-axis) using identical RNA samples. Each circle corresponds to one gene of our custom gene panel measured on both platforms. Curves in red indicate the local regression *(LOESS)* fit between the two profiles, and shades in red give 95% confidence interval of the fitting. The grey vertical line indicates the detection limit of microarrays and horizontal line the arbitrary detection limit of less than one read per sample. **(B)** Gene expression profiles of the same samples probed by AmpliSeq-RNA (y-axis) and by conventional quantitative RNA sequencing (x-axis). Curves and shades in red, like in **(A)**, give the LOESS fit and 95% confidence interval of the fitting. Horizontal and vertical dash lines indicate the detection limit and transcripts below one count per gene are considered as being absent. Time points of each panel are indicated in the top bar and the correlation coefficient is displayed in the top left corner of each diagram.

**Figure 3:**
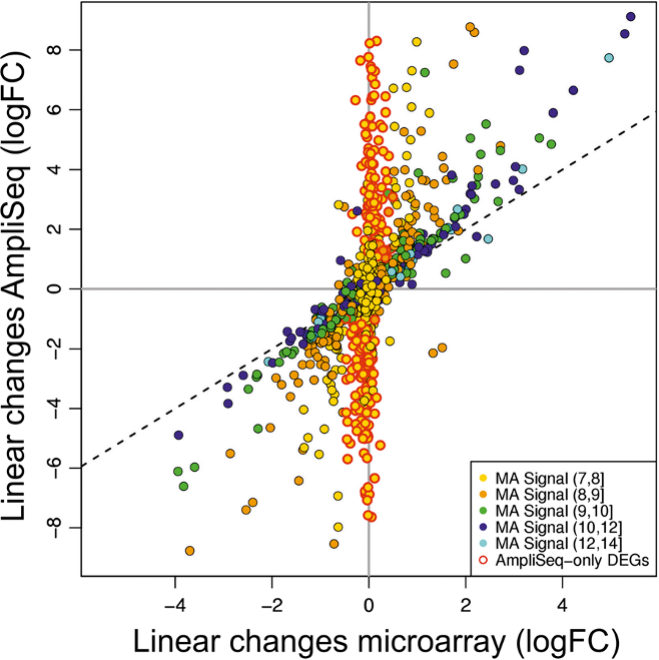
**Comparison of linear gene expression changes of differential called by AmpliSeq-RNA (y-axis) and by microarray (x-axis).** Each dot corresponds to a gene with an absolute log fold-change larger than one and a p-value smaller than 0.05 using data from all time points. Yellow dots with a red outline correspond to genes (n = 237) for which significant differential gene expression changes are only captured by AmpliSeq-RNA. Color codes corresponding to microarray (MA) log2 expression levels are shown in the legend insert. Note that DEGs with moderate to high expression are captured by both technologies with comparable change factors.

Next we compared the read-frequencies measured by AmpliSeq-RNA with equivalent data from Illumina microrarrays [20] or from quantitative RNA sequencing [21] at day 0, 10, 20 and 60 (Figure 2). We selected an average amplicon length of about 100 nucleotides matching the median read length of the Ion-Torrent Proton sequencing technology. As a result, the number of reads per amplicon is directly proportional to transcript abundance circumventing the need for data normalization. In contrast, results obtained by conventional quantitative RNA sequencing require length normalization and are expressed as RPKM (reads per kilo base per million reads) values. In addition, uneven read coverage along a given transcript can complicate precise quantification. Fluorescence-based microarray technology suffers from low sensitivity as result of background noise. Nevertheless, we included this technology for benchmarking with published data. Above the noise level of 8 on log_2_ scale we found an almost linear relation of AmpliSeq-RNA and microarray data at a confidence level of 95% across all time points. Genes below this noise level are not detected resulting in a sigmoid curve (Figure 2A). The correlation coefficient of 0.77 for AmpliSeq –RNA and microarrays is similar to the correlation of r = 0.73 found in a comparison between microarrays and conventional, quantitative RNA-sequencing [19]. To confirm expression of all transcripts detected by AmpliSeq-RNA we profiled the same samples by conventional strand-specific RNA sequencing as an independent technology with comparable sensitivity. We obtained a nearly linear relation between the two datasets with an average correlation coefficient of r ≈ 0.87 for all time points (Figure 2B).For experimental applications in areas such as translational medicine or biomedical research the ability to measure dynamic transcriptional responses to stimuli is often more relevant than the determination of absolute expression values. We addressed this point by comparing detection of differential gene expression by AmpliSeq-RNA and microarrays. We selected differentially expressed genes (DEGs) from all time-points with an absolute log fold-change larger than one and a p-value smaller than 0.05 (Figure 3). Less abundant DEGs (n = 237) fulfilling the filter criteria above are only reported by AmpliSeq-RNA thereby extending the scope of the analysis.

### Biological significance of AmpliSeq-RNA data

Despite single molecule sensitivity of deep sequencing, a specific set of about 50 genes is undetected at each time point (Table 1). Five genes with preferential expression in neuronal tissues are absent in all time-points. This finding shows indirectly that absent calls are related to lack of gene expression rather than to technical issues. Transcripts of the FOXA signaling network for instance are not detected in iPSCs. The forkhead-box-domain transcriptional regulator family has about forty functionally distinct member genes and plays pivotal roles in mammalian development [22]. Apart from being a physiological master regulator, FOXA1 is a potent stimulator of cell proliferation in tumors [23, 24]. Likewise, transcripts activated by the mitogen-activated protein (MAP) kinase signaling network are not detectable in iPSCs cells. MAP-kinase signaling has pleiotropic functions controlling inflammation, cell cycle, cell death, development, cell differentiation, senescence or tumorigenesis in specific cell types [25]. Suppression of FOXA and MAP-kinase signaling is probably crucial for maintenance of stem cell pluripotency and homeostasis. Once cardiomyocyte differentiation is completed genes belonging to the *RAS*- and *JNK*- signaling cascades are suppressed. The *RAS* pathway is an important regulator of cell differentiation and a point of convergence for multiple signaling cascades [26]. Janus kinase signaling is expected to induce further cell proliferation and apoptosis [27]. Suppression of the signal transduction by the pathways briefly described is consistent with biological function suggesting faithful performance of AmpliSeq-RNA transcript imaging.

**Table 1:** **Specificity of AmpliSeq-RNA: Number of undetected amplicons at each time point**

Day 0	73
Day 10	49
Day 20	55
Day 60	83
Absent	5

The numbers refer to undetected genes in the entire panel of 917 transcripts. 5 transcripts are absent considering all time points.

**Figure 4:**
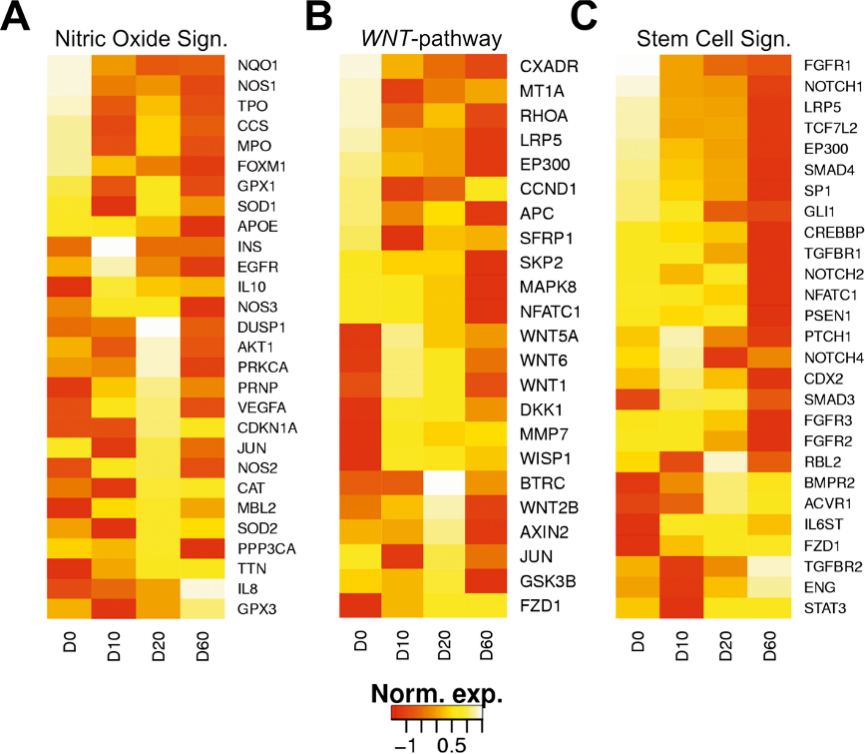
**Expression patterns of selected genes involved signaling pathways for cell plasticity, growth and differentiation: (A) nitric oxide signaling (NOS) pathway (28 genes); (B) WNT pathway (23 genes); (C) stem cell signaling (27 genes).** Relative transcript abundance normalized per gene across four time points are visualized with ‘cascade plots’. The order of genes from top to bottom was generated according to expression at each time point. The diagrams were scaled to the same size. Transcript abundance is color coded from low (red) to high (yellow).

In order to detect transcriptional re-programming during differentiation, we chose three master regulator networks involved in eukaryotic development. The *NOS*-signaling pathway has protective properties such as detoxification and is regulated by nitric oxide the only gas activator of eukaryotic development [24] . The family of nitric oxide synthases catalyzes production of protective nitric oxide from L-arginine following signal transduction to downstream pathways. The *WNT* and stem signaling cascades are functionally closely related and regulate several aspects of development including organogenesis, midbrain development as well as stem cell proliferation [28]. *WNT* signals control cell fate determination, cell movement and tissue polarity. Loss-of-function mutations and epigenetic silencing in *WNT* signaling genes occurs in a variety of human cancers. Notch and the fibroblast and transforming growth factor receptors are key regulators of the morphogenetic stem cell signaling network. This pathway is implicated in the maintenance of tissue homeostasis by regulating self-renewal of normal stem cells as well as proliferation and differentiation of progenitor cells. To demonstrate developmental modulation of these pathways we monitored specific sets of about 25 transcripts for each of them. For *NOS*, *WNT* and stem cell signaling essentially all member genes were found to be differentially expressed during differentiation (Figure 4). Regardless of the pathway, all genes with high expression in stem cells are repressed in the terminal cardiomyocyte stage and *vice versa*. Four *WNT* genes present in our panel are only expressed in the transition phase at day 10 and 20 reflecting the role of *WNT*-proteins in tissue differentiation and cytoskeleton plasticity. We conclude that differential gene expression measured by AmpliSeq-RNA data matches results obtained from genome-wide transcript profiling.

## Discussion and conclusions

The current trend to study subsets of genes rather than genome-wide gene expression requires appropriate technologies. Apart from significant cost-reduction, data interpretation is less complicated and more conclusive due to focus on relevant data points. The current version of the microfluidics based Fluidigm technology [1] measures expression of 96 custom genes in 96 samples by qPCR. Despite high sensitivity, throughput and dynamic range are suboptimal. In addition, a significant hardware investment is involved. Standard quantitative RNA sequencing comes with single molecule sensitivity and a dynamic range of at least five orders of magnitude depending on instrument platform and multiplexing. High-throughput sample processing is currently restricted by the largely manual workflow of most platforms, expenses and lavish data processing. We argue that AmpliSeq-RNA can potentially close these gaps. We demonstrate parallel amplification of 917 transcripts in a single tube with comparable efficiency which we consider sufficient for most biological and medical research applications. We performed parallel sequencing of 12 barcoded samples to explore the full dynamic range of the Proton-technology delivering about 12 million reads per sample under optimal conditions. For most applications, a profile based on 1 million reads per sample is sufficient for discovery of significant biological processes. Such a high-level analysis allows multiparallel analysis of up to 150 samples in a single run. The short three-hour run time of Proton sequencers permits completion of two experiments per day within normal working hours. The low amount of input RNA ranging from 500 picogram to 5 nanogram allows analysis of biopsy derived tissue samples and the small amplicon size of 100 base pairs is compatible with partially degraded FFPE-tissue [12]. The steadily increasing acceptance of human primary- or iPSC derived three-dimensional *in vitro* tissue models as surrogate for clinical responses is another area where only small amounts of RNA are available from spheroid body cultures. Apart from multiplexing options with respect to sample number and analysis content, we consider sequence based quantification as an important feature of AmpliSeq-RNA. Pooling of up to 1’000 primer pairs will likely result in off-target amplification which cannot be detected by fluorescence based detection methods. In fact, we found experimentally that about 3% of all reads do not map to the amplicon sequence pool probably due to unspecific off-target amplification. In contrast to conventional RNA sequencing that employs genomic read mapping, AmpliSeq-RNA considers only reads matching the correct target sequence for quantification resulting in elimination of off-target born amplicons. Defining the number of mismatches in the sequences used for mapping allows detection of genetic variations such as single-nucleotide polymorphisms (SNPs) and point mutations in the transcript provided the amplicon covers such polymorphic stretches. Along the same lines, targeting of conserved sequence stretches flanking polymorphic regions in closely related homologs of gene families allows quantification of each transcript variant. Finally, we expect that amplicon based mRNA quantification can be performed on any deep sequencing instrument with sufficient data output. However, semiconductor sequencing is by far the fastest method with three hours run time and automated read quantification on the Ion-Torrent system server.

## Methods

We have compiled a panel of 917 transcripts derived from more than 100 human signaling cascades and signaling networks. These were submitted via web interface for primer design and probe pool synthesis using proprietary algorithms (https://www.ampliseq.com/browse.action). For data analysis, a canonical sequence reference file and a plug-in for the Ion-Torrent server are provided by the supplier of the PCR-primer pool (Life Technologies, Carlsbad, USA).

### Samples for validation

We have previously analyzed transcriptional changes during differentiation from human iPSCs to beating cardiomyocytes at multiple time points [14]. The human cells were of commercial origin and are not subject to ethical committee approval. We selected triplicate RNA samples from day 0, 10, 20 and 60 for analysis to confirm transcriptional changes measured by whole genome microarray analysis as reference and check for sample integrity according to the published protocol using the same microarray platform (HumanHT-12 v4.0 Beadchip, Illumina Inc., San Diego, USA).

### Amplicon library construction

10 ng of total RNA from each time point and biological replicate were reverse transcribed to cDNA by poly-A-priming followed by PCR pre-amplification (15 cycles) according to the protocol supplied with the Ion AmpliSeq™ RNA Library Kit (Life Technologies, Carlsbad, USA, Catalog number 4482335). After primer digestion, adapters and molecular barcodes were ligated to the amplicons followed by magnetic bead purification. This library was amplified, purified and stored at -20°C. Amplicon size and DNA concentration were measured using an Agilent High Sensitivity DNA Kit (Agilent Technologies, Waldbronn, Germany) according to the manufacturer’s recommendation.

### Semi-conductor sequencing

5 picomoles of each of the 12 samples (4 time points; 3 biological replicates) were barcoded and pooled for emulsion PCR (ePCR) on Ion Sphere Particles (ISPs) using the Ion PI Template OT2 200 Kit v3 using the Ion OneTouch 2 Instrument. Following automated Ion OneTouch ES enrichment of template-positive ISPs samples were loaded on an Ion Proton PI chip v2 and sequenced on an Ion Proton instrument using the Ion PI sequencing 200 kit v3 (Life Technologies, Carlsbad, USA).

### Quantitative RNA sequencing

The same samples as above were sequenced using commercial kits and protocols provided by Life Technologies, Carlsbad, USA. Briefly, 1.5 μg of total RNA was used to construct the strand-specific mRNA-Seq libraries using the SENSE mRNA-Seq Library Prep Kit (Lexogen, Vienna, Austria). Template preparation and Proton sequencing was done according to the manufacturer’s instructions (Life Technologies, Carlsbad, USA).

### Read mapping and quantification

Reads were mapped and quantified using the program TMAP (Torrent Mapping Alignment Program; http://mendel.iontorrent.com/ion-docs/Technical-Note---TMAP-Alignment_9012907.html) which is based on different alignment algorithms [29–32]. The program runs automatically on the Ion-Torrent Server following completion of sequencing. Reads are mapped to the amplicon sequences of the panel resulting in fast data processing, quantification with concomitant elimination of unspecific amplicons (typically ~3% of all reads).

### Detection of differentially expressed genes

Differentially expressed genes (DEGs) were identified from microarray data based on linear models using the *limma* package in Bionconductor (http://www.bioconductor.org). We applied three data modelling approaches to AmpliSeq-RNA with increasing complexities: 1) log_2_ transformation (adding an epsilon of 1E-3 to all read counts) 2) *voom* (*limma* package) modelling of mean-variance relationship and 3) *edgeR* based on negative binomial distribution [33]. Differential gene expression values determined by three approaches are virtually identical (R^2^ > 0.99).

### Supporting data

The gene expression raw data are deposited in the GEO database (http://www.ncbi.nlm.nih.gov/geo/;Accession number: GSE58737).
